# Dentofacial Deformity Caused by Bulky Osteochondroma: Report of an Unusual Case and the Importance of Cone Beam Computed Tomography

**DOI:** 10.2174/1874210601711010237

**Published:** 2017-04-28

**Authors:** Weeraya Tantanapornkul, Kittipong Dhanuthai, Phonkit Sinpitaksakul, Chumpot Itthichaisri, Paksinee Kamolratanakul, Vacharee Changsirivatanathamrong

**Affiliations:** 1 Department of Oral Diagnosis, Faculty of Dentistry, Naresuan University, Phitsanulok, Thailand; 2 Department of Oral Pathology, Faculty of Dentistry, Chulalongkorn University, Bangkok, Thailand; 3 Department of Radiology, Faculty of Dentistry, Chulalongkorn University, Bangkok, Thailand; 4 Department of Oral and Maxillofacial Surgery, Faculty of Dentistry, Chulalongkorn University, Bangkok, Thailand

**Keywords:** Dentofacial deformity, Condyle, Conebeam CT, Osteochondroma, Tomography

## Abstract

**Introduction:**

Osteochondroma of mandibular condyle is a rare benign tumor.

**Case Report:**

This case report described clinical, radiographic features, differential diagnosis, histopathologic correlation and treatment of condylar osteochondroma.

**Conclusion:**

Conebeam computed tomography (CBCT) is an alternative modality to CT or MRI that should be performed in all cases of suspected osteochondroma of the mandibular condyle.

## 1 INTRODUCTION

Osteochondroma is one of the mostly found axial skeleton benign tumors. However, it is rarely found in maxillofacial area. Mandibular osteochondromas occur in coronoid process, condyle, ramus, body, angle, and symphysis regions [1-5]. Mandibular condyle is most frequently affected [1]. It is a cartilage-capped exophytic lesion. Trauma and inflammation have been implicated as predisposing factors [6, 7]. Clinical features of condylar osteochondroma include facial asymmetry, prognathic deviation, posterior crossbite, condylar morphology changes [2,8], which may resemble those seen in patients with temporomandibular joint disorders (TMD).

Conebeam computed tomography (CBCT) is an acceptable alternative for the evaluation of mandibular condyle [12]. Treatment of condylar osteochondroma is surgical excision of the lesion with mandibular condyle [1, 9-11]. The purpose of this case report was to describe clinical, radiographic features, differential diagnosis, histopathologic correlation and treatment of condylar osteochondroma.

## 2 CASE REPORT

**Fig. (1):**
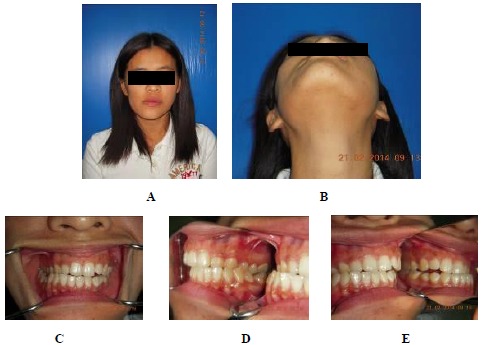
**A, B** Extraoral examination showing facial asymmetry with the chin point deviated to the right. **C, D, E** Unilateral posterior crossbite on the right side and open bite on the left side.

A 29 years old female presented with facial asymmetry, pain in the left temporomandibular joint (TMJ) and headache. Her past medical history revealed that she had a trauma to her chin 15 years ago and from that time on, a slowly progressing facial asymmetry and limitation in the mouth opening began. The clinical examination revealed an obvious facial asymmetry with the chin point deviated to the right. Non tender bony hard swelling on left TMJ was found, including reduced jaw movement, unilateral posterior crossbite on the right side, open bite on the left side and limited mouth opening (28 mm). Clicking sound was observed on opening and closing the mouth (Fig. **1**).

**Fig. (2):**
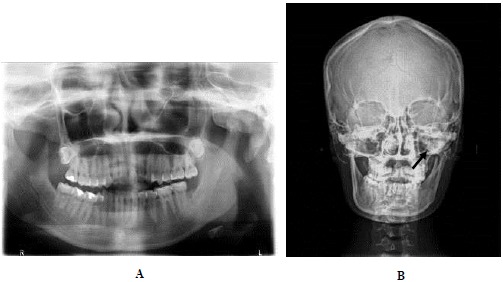
**A**. Panoramic radiograph showed a radiopaque area on the left condylar region with similar density to the adjacent bone. **B**. PA skull showed a well defined bony overgrowth extending medially from the left condyle, facial asymmetry and deviation of mandible to the right side.

**Fig. (3):**
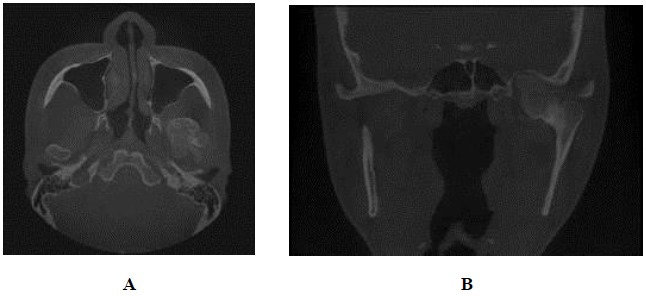
**A, B** Axial and coronal CBCT images showed a large bony mass arising from the left mandibular condyle extending anteriorly, posteriorly, medially and superiorly to the temporal bone. The lesion density was continuous with the structures of mandibular condyle.

Panoramic radiographic examination showed an irregular radiopaque lesion in association with the left condylar head. It had similar density to the adjacent bone. Postero-anterior (PA) skull showed a well defined bony overgrowth extending medially from the left condyle, facial asymmetry and deviation of mandible to the right side (Fig. **2**). CBCT showed a large bony mass arising from the left mandibular condyle extending anteriorly, posteriorly, medially and superiorly to the temporal bone. The lesion density was continuous with the structures of the mandibular condyle (Fig. **3**).

**Fig. (4):**
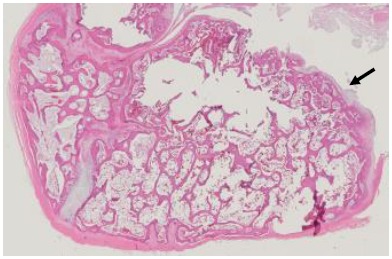
Histopathologic examination revealed small and large cancellous bones covered in some parts by cartilaginous cap (arrow).

Surgical treatment plan called condylectomy and extraoral vertical ramus osteotomy were performed under general anesthesia. Histopathologic examination revealed small and large cancellous bones covered in some parts by cartilaginous cap. This finding was consistent with osteochondroma, which was the final diagnosis of this lesion (Fig. **4**).

After the operation, symptoms including TMJ pain and headache disappeared. The patient almost re-attained facial symmetry. The contralateral posterior crossbite was resolved. In addition, the mouth opening was normal (38 mm) with slight deviation. For the correction of the midline deviation and occlusion, adjunctive orthodontic treatment was suggested.

## 3 DISCUSSION

Although osteochondromas are one of the most common benign bone tumors, they are relatively rare in the maxillofacial region. Early investigation and diagnosis are essential in order to provide proper treatment, which may affect the patient’s quality of life. Condylar osteochondromas are usually situated on the anteromedial surface of the condylar head [11], however, the lesion was found on superior and posterior surface of the condyle in this patient. The occurrence of these tumors in the condyle tends to support the theory of aberrant foci of epiphyseal cartilage on the surface of the bone [4]. It is accepted that stress in the tendinous insertion region of lateral pterygoid muscle, where focal accumulations of cells with cartilaginous potential exist, leads to the formation of these tumors. The possible reason for the rare occurrence of osteochondroma in the maxillofacial skeleton is the intramembranous development of these bones [12]. This may also explain the occurrence of osteochondromas in the coronoid process as the result of stress by the tension of temporalis muscle. Although the etiology of these lesions is still controversial, neoplastic, developmental, reparative, and traumatic etiologies have been proposed [8, 9]. For the present case report, the history of trauma might be the possible cause of osteochondroma.

Panoramic radiograph is mainly used for screening purposes, however, the high rate of false-positive results for TMJ pathology evaluation suggests that if the clinical finding is positive, more advanced techniques such as CT and/or Magnetic Resonance Imaging (MRI) are indicated, even if the panoramic findings are negative. CBCT is an acceptable alternative to medical CT for the evaluation of osseous abnormalities of the mandibular condyle with almost equal high sensitivity and specificity [13]. CBCT overcomes conventional radiography such as panoramic or posteroanterior images, which could be demonstrated by only two-dimensions that cause anatomical superimposition. CBCT brings to clinicians the possibility of evaluating complex cases in the maxillofacial regions and giving information that leads to more accurate and specific diagnosis of some TMJ pathological conditions including osteochondroma. It provides geometrically accurate images and excellent spatial resolution. Multiplanar reformatting and data manipulation allow maxillofacial pathologies to be assessed in sagittal, coronal, and axial planes. In addition, 3-D surface rendered reconstruction of the maxillofacial structures could be demonstrated, important for the differential diagnosis and treatment planning.

Osteochondroma should be differentiated from lesions involving mandibular condyles or TMJ complex. Condylar hyperplasia mimics osteochondroma clinically, however, they are different on radiographic findings. Condylar hyperplasia shows a proportionate growth of the involved condyle as a whole, with elongation of the neck of condyle. Osteochondroma, on the other hand, shows an exophytic growth from condyle. Conventional radiographs are difficult to differentiate osteomas from osteochondroma. Osteomas of the condyle may grow as a sessile or pedunculated mass from condyle [14]. Multiple radiopaque lesions in the jaws are indicative of osteomas. Osteomas are slow growing and present in a much smaller size as compared to osteochondroma [15]. The histologic criteria for the diagnosis of an osteochondroma include chondrocytes of the cartilaginous cap arranged in clusters in parallel oblong lacunar spaces similarly to those of normal epiphysial cartilage. The histologic orientation is suggestive of a benign lesion [12].

Treatment for osteochondroma sometimes requires adjuvant orthognathic correction [7]. Improper case selection for partial/conservative condylectomies can lead to recurrence of lesion or rarely malignant transformation. Conservative osteotomy may require additional occlusal and gnathic asymmetry correction, which include orthodontic treatment and selective osteotomies for facial asymmetry correction [13].

## CONCLUSION

Osteochondroma of the mandibular condyle is a rare benign tumor. However, it should be considered in the differential diagnosis of bony masses in the TMJ region. Panoramic and/or other conventional radiographs can be considered as screening modalities. CBCT is an alternative modality to CT or MRI that should be performed in all cases of suspected osteochondroma of the mandibular condyle.
